# The Effectiveness of Internal Maxillary Sinus Elevation Using Controlled Hydrodynamic or Pneumatic Pressure: An Ex-vivo Experimental and Preliminary Animal Study

**DOI:** 10.7759/cureus.26711

**Published:** 2022-07-10

**Authors:** Yazan Hudaifa, Mohammad Y Hajeer, Mohammed Monzer Alsabbagh, Mhd Ammar Kouki

**Affiliations:** 1 Department of Periodontology, University of Damascus Faculty of Dentistry, Damascus, SYR; 2 Department of Orthodontics, University of Damascus Faculty of Dentistry, Damascus, SYR

**Keywords:** experimental animal study, animal study, controlled pneumatic pressure, controlled hydrodynamic pressure, sinus membrane elevation, maxillary sinus

## Abstract

Objective

The aim of this experimental study was to test the possibility of applying internal sinus elevation techniques using controlled hydrodynamic or pneumatic pressure and evaluate their elevation effectiveness.

Materials and methods

A device was specially designed for this study and was used to elevate the sinus membrane internally in 12 halves of freshly slaughtered sheep heads. The sample was divided into two groups randomly according to the type of controlled pressure applied (hydrodynamic or pneumatic). The elevation height of the membrane was measured in addition to investigating the presence or absence of perforation.

Results

The maxillary sinus membranes started to be elevated at an average pressure value of 21.6 ± 7.5 millibars (mbar) when hydrodynamic pressure was applied, and at an average pressure value of 23.3 ± 8.1 mbar when pneumatic pressure was applied. The mean values ​​of elevation height after applying the controlled hydrodynamic and pneumatic pressure were 13.00 ± 2.76 and 10.33 ± 3.88 mm, respectively. No perforations occurred in either of the groups.

Conclusions

The use of a controlled hydrodynamic or pneumatic pressure, which is appropriate for the characteristics of the maxillary sinus membrane in the process of internal elevation, is effective, and it yielded an amount of lifting similar to that we get when using the external elevation.

## Introduction

The compensation of the upper posterior teeth is considered one of the most common cases encountered in dentistry as 13 million American adults, approximately 17.5% of the adult population, lose all their upper posterior teeth [1]. This procedure has many challenges when performed in the upper posterior region [2], such as a reduction in the height of the alveolar bone following the wastage of teeth resulting from the phenomenon called extensive maxillary sinus pneumatization [3]. Moreover, the alveolar bone width decreases at a faster rate than any other area in the jaws after teeth loss [4]. In order to combat these challenges, several techniques have been proposed for the purpose of elevating the maxillary sinus membrane through the lateral window (external sinus lift), or through alveolar bone (internal sinus lift) [5], i.e., to increase the height of the superior alveolar bone, and placing implants in an appropriate bone density [2]. The maxillary sinus lifting alone, or combined with dental implantation, has recently become a procedure with predictable results [2]. Sinus lifting operations have become more common with the advent of cone-beam CT (CBCT) [6]. In order to perform this treatment, an understanding of the radial and clinical anatomy of the maxillary sinus is required [7].

Nonetheless, a sinus infection is a very common complication of this procedure, and it is possible that this infection not only results in the failure of sinus elevation but may also threaten the patient's life [7]. The maxillary sinus membrane perforation is the most common complication during the maxillary sinus lifting, and the presence of bone septa and tooth apexes that penetrate the bottom of the sinus may increase the possibility of this occurring [8,9]. This perforation, in turn, increases the possibility of maxillary sinus infection three to seven days after surgery, and this may lead to the failure of elevation. Furthermore, it may develop into chronic sinusitis, and the infection could spread to the orbit or the brain [7]. Surgical techniques for internal sinus lift depend mainly on the fracture of the sinus floor using different methods with an emphasis on safety and effectiveness [10]. After the study by Summers, which tested the sinus floor elevation by using osteotomes [11], many researchers have aimed to test new techniques, such as in the studies by Sotirakis et al. [12], Kim YK et al. [13], and Kim JM et al. [14]. These three studies used hydrodynamic pressure in order to effectively elevate the maxillary sinus membrane through the alveolar bone. Recently, the properties of the maxillary sinus membrane have been thoroughly studied in order to develop a more controlled approach, such as the one proposed by Pommer et al. [15], and Troedhan et al. [16].

Currently, there are many animal models available to test different maxillary sinus lift techniques. The first research that aimed to study the morphology of the maxillary sinus in sheep was performed by Valbonetti et al. [17]. They used CBCT in order to define its descriptive anatomy and compare it with the maxillary sinus in humans. They reported that the sheep is the ideal animal model for testing maxillary sinus elevation techniques, due to its widespread availability, in addition to the anatomical similarity with the human maxillary sinus [17]. Radiological studies using CBCT on the healthy sinus of the sheep have provided accurate morphological information. The classic anatomical textbook has reported that the maxillary sinus and palatine sinus of the sheep is widely communicating, and separated by an infraorbital canal that arises from the bony floor that is different from humans [17]. The maxillary sinus in sheep extends from the third premolars region, through the third molar to the extraoral space [17].

Recently, a special device for the internal maxillary sinus left procedures was developed at the Faculty of Dentistry in cooperation with a research team at the Mechanical Engineering Faculty at the University of Damascus, Syria. This device was designed and manufactured for using the pneumatic pressure of the dental unit (approximately 4 bar) in the internal maxillary sinus lift procedures by converting that pressure to a low and controlled hydrodynamic or pneumatic one, suitable for mechanical properties of the maxillary sinus membrane. This device has not been tested on animals. In light of this, the objectives of this preliminary study were threefold: (1) to test the appropriate method for applying the internal maxillary sinus elevation technique using either a controlled hydrodynamic or pneumatic pressure; (2) to measure the resulting elevation height; and (3) to detect any possible perforations in the raised membrane.

## Materials and methods

Study design and setting

This experimental animal study was carried out at the Department of Periodontology, Faculty of Dentistry at Damascus University. Approval for this study was obtained from the Local Research Ethics Committee of the Faculty of Dentistry, Damascus University (UDDS-582-24082020/SRC-3075). The authors did not receive any support or funding from any organization for the submitted study.

The collected sample

The study sample consisted of 12 halves of sheep (Awassi breed) heads that were freshly slaughtered for commercial purposes. The exclusion criteria were the presence of defects or lesions in the maxillary sinus that could be detected on CBCT images, and the presence of a maxillary sinus less than 20 mm in height, which could complicate the monitoring and the measuring of the elevation process.

The controlled internal maxillary sinus elevation device (CIMSED)

**Figure 1:**
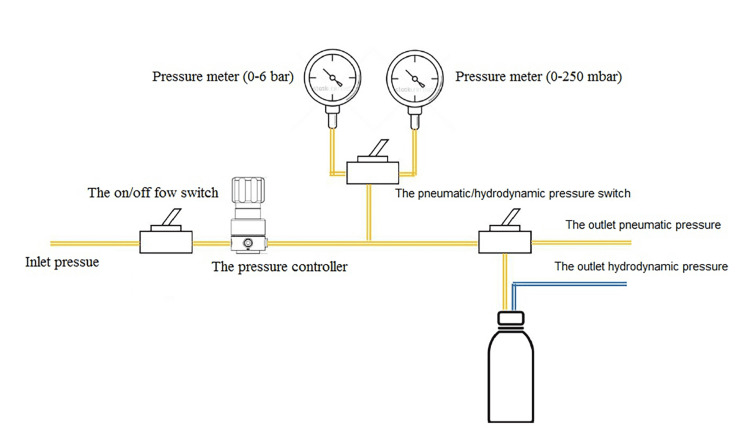
The working principle of the controlled internal maxillary sinus elevation device (CIMSED) This diagram was created by the authors of this paper

The controlled internal maxillary sinus elevation device (CIMSED) was used to apply a regulated hydrodynamic or pneumatic pressure to the sinus membrane (Figure 1), starting at 10 millibars (mbar) based on Troedhan et al.'s study [16]. Then, this pressure was raised gradually, in order to achieve the desired elevation. The pneumatic pressure of the dental unit was about 4 bar and was delivered to the CIMSED device through an 8-mm nylon tube.

**Figure 2:**
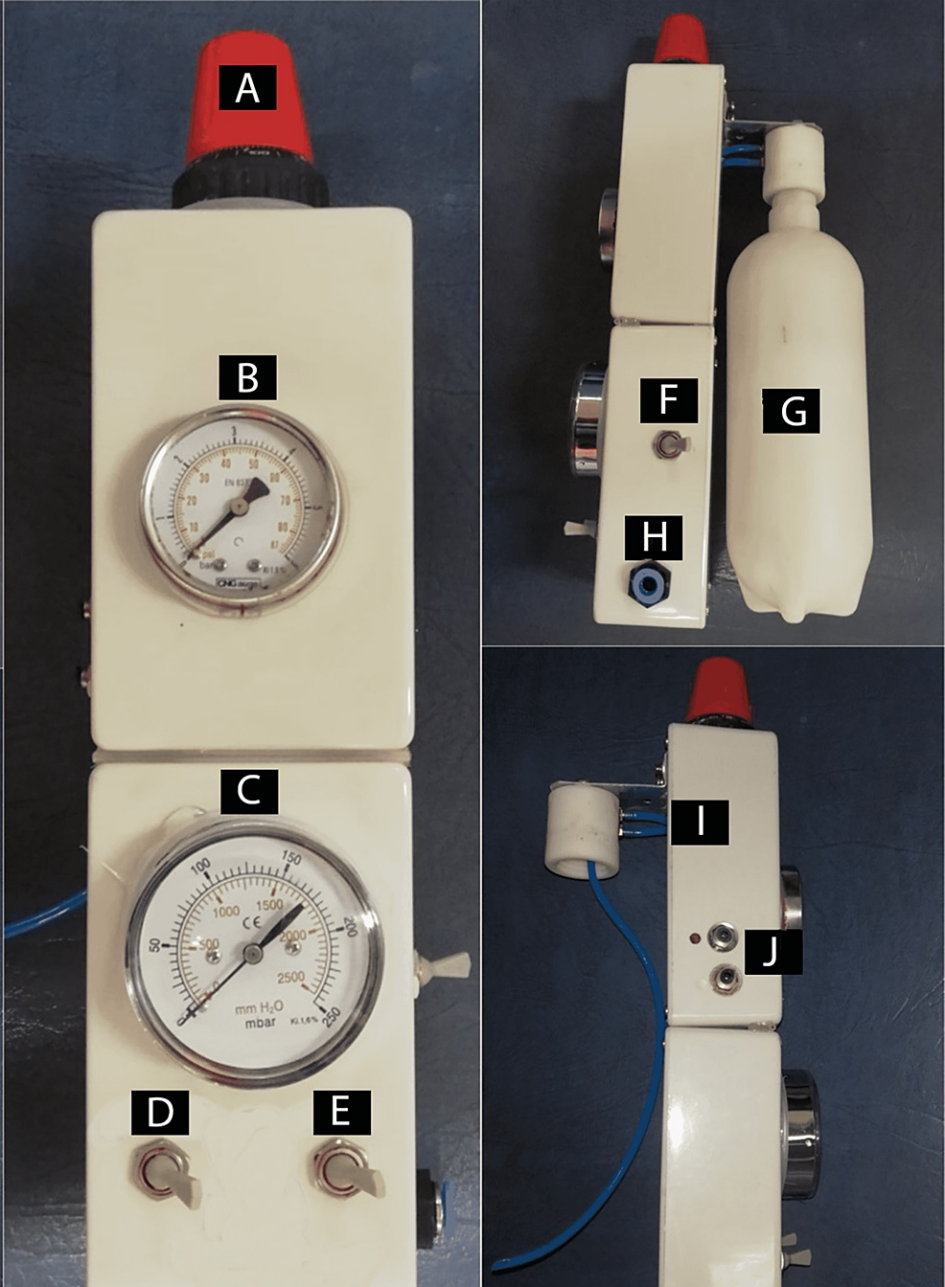
The controlled internal maxillary sinus elevation device (CIMSED) components A: The pressure controller. B: The pressure meter with a range of 0-6 bar. C: The pressure meter with a range of 0-250 mbar. D: The pneumatic/hydrodynamic pressure switch. E: The on/off flow switch. F: The pressure meter switch. G: The plastic package. H: The inlet pressure input. I: The 3-mm nylon tubes. J: The controlled pneumatic/hydrodynamic pressure output The components of this system (CIMSED) were all manufactured/developed locally without any assistance from any specific commercial manufacturer

This pressure was controlled by a pressure controller (Figure 2A) to regulate it to a pressure suitable for the characteristics of the maxillary sinus membrane. The CIMSED device was equipped with two pressure meters to measure the outlet pressure, whether it was pneumatic or hydrodynamic: the first with a range of 0-6 mbar (Figure 2B) and the second with a range of 0-250 mbar (Figure 2C). The purpose of having two pressure meters was to expand the pressure reading range while ensuring high accuracy at small values (mbar rank). There was a special switch for switching between the two gauges (Figure 2F). The CIMSED was provided with a pneumatic/hydrodynamic pressure switch (Figure 2D). When the switch was set on the pneumatic pressure mode, the controlled pneumatic pressure was pushed out from its output (Figure 2J). When the switch was put on the hydrodynamic pressure mode, the regulated pneumatic pressure was transferred to the saline-filled package through the 3-mm nylon tube (Figure 2I), until the pressure within it became equal to the regulated pressure, causing the saline to be pushed out from it through another 3-mm nylon tube (Figure 2I), according to the readable controlled pressure on the pressure meter. The outlet pressure (Figure 2J) could be stopped by the on/off flow switch (Figure 2E), when the observation was needed or when the required height of the maxillary sinus was reached. Finally, the controlled outlet pressure was conveyed to the maxillary sinus membrane by a 3-mm silicone tube without loss or dispersion.

The surgical procedures

**Figure 3:**
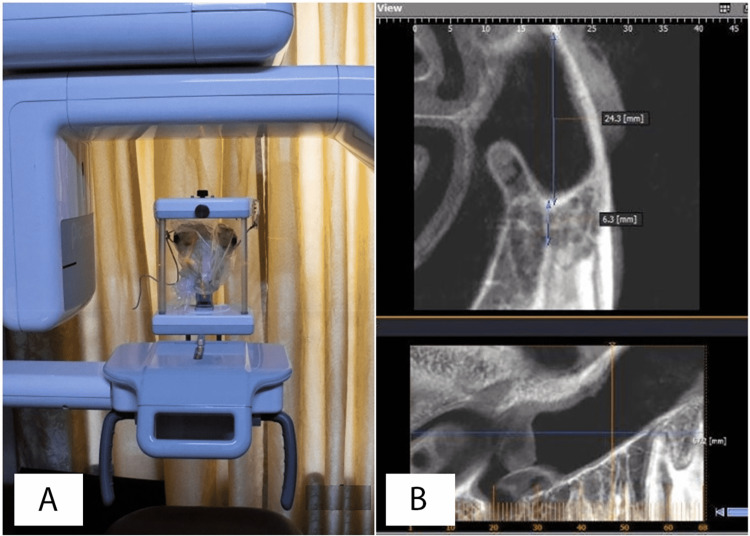
A: The CBCT scan using Picasso® Pro CBCT system. B: The determination of the appropriate location of elevation CBCT: cone-beam computed tomography

Before performing the surgical procedures, a CBCT scan was taken using a Picasso® Pro CBCT system (Vatech, Seoul, South Korea) for each head (Figure 3A). The CBCT scans were used to investigate the integrity and the height of the maxillary sinus, as well as the height of the posterior alveolar bone. Using this imaging technique was essential to determine the appropriate location of elevation (the height of the bone below the base of the maxillary sinus should be from 5 mm to 12 mm, Figure 3B).

**Figure 4:**
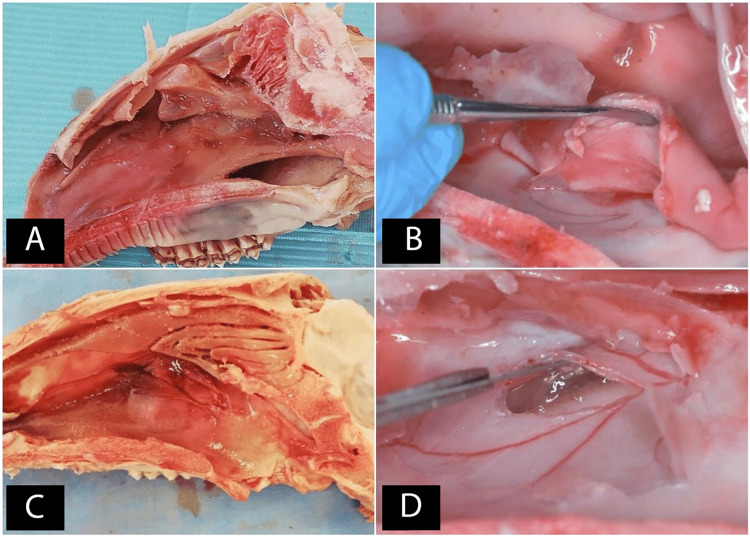
A: The sheep's half head (lateral view). B: Dissecting the nasal mucosa. C: After removing the lower and middle nasal concha. D: Preparing the monitoring window

All the sheep heads were put in saline immediately after the slaughter to avoid postmortem drying, and the surgical procedures were started within a maximum of two hours after the slaughter. This was because gross changes may start two hours after death [18]. Although the livor mortis may begin after half an hour, the previously mentioned timeframe was adopted since the sinus membrane is poorly vascularized [18]. Initially, a sagittal cutting of each head was performed with an iron saw (Figure 4A), and the nasal mucosa was dissected by a periosteum elevator and number 15 (N15) surgical blades (Figure 4B). After that, the lower and middle nasal conchae were removed using surgical scissors and elevators, in order to expose the maxillary sinus from the inside through its medial wall (Figure 4C). This exposing was done by making a bony window (similar to the window used when performing external maxillary sinus elevation), using a 2-mm spherical diamond bur mounted on a micro-motor handpiece connected to the surgical motor (the speed was 800 rounds per minute), with cooling by the saline, and then by making a hole in the exposed membrane (Figure 4D).

Having analyzed the CBCT scans and determined the appropriate location of the elevation across the posterior upper alveolar ridge (Figure 3B), the internal maxillary sinus elevation was done, starting from extracting the tooth in the appropriate place (if present) with elevators and the posterior upper molar clasps. The Crestal Approach Sinus kit (CAS kit, Osstem Implant Co, Seoul, South Korea) drills were used to prepare the proposed implant bed (the location of the elevation), utilizing a micromotor handpiece connected to a surgical motor (the speed was 500 rounds per minute), with cooling by saline.

**Figure 5:**
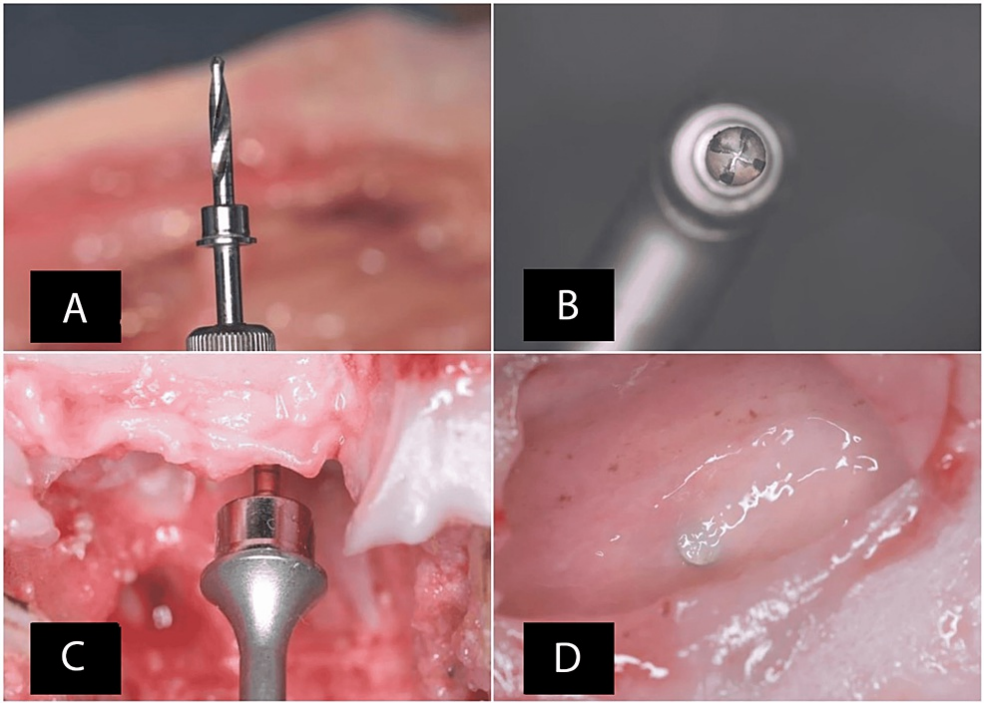
A: The 2.2-mm twisted drill. B: The 3.1-mm drill. C and D: Examining the integrity of the maxillary sinus membrane and the bone height using the depth gauge provided by the Crestal Approach Sinus kit (Osstem Implant Co)

The precise location of the preparation was determined by using the guide drill with a diameter of 2 mm. The primary preparation was performed by using the 2.2-mm twisted drill (Figure 5A), until 2 mm less than the inferior wall of the sinus, through the use of an appropriate stopper. The preparation was widened by using the 2.8-mm CAS drill, and, at the same time, was extended 1 mm towards the inferior wall of the maxillary sinus. Finally, the preparation was extended to the inferior wall of the sinus using the 3.1-mm CAS drill (appropriate to the diameter of the tube connected to the CIMSED, Figure 5B). The CAS drills were used to make sure that the inferior wall of the sinus was safely broken, without damaging the lining membrane, by lifting it slightly across the cone-shaped bone resulting from the preparation [13] (Figure 5B). Before proceeding with the next step, the integrity of the maxillary sinus membrane and the bone height was examined, using the depth gauge provided by the CAS kit (Figures 5C, 5D).

**Figure 6:**
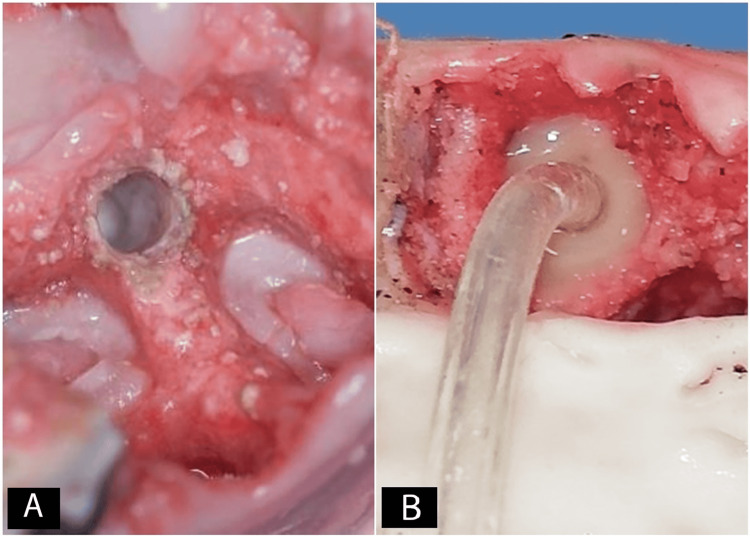
A: Implant bed. B. Inserting the silicone tube that is connected to a controlled internal maxillary sinus elevation device in the prepared implant bed until it reached the membrane of the maxillary sinus, and sealing it using the flowable composite

At this stage, the silicone tube connected to CIMSED was inserted in the prepared implant bed (Figure 6A) until it reached the membrane of the maxillary sinus, and it was sealed with the flowable composite to ensure adequate isolation, as well as prevent air or saline leakage (Figure 6B).

**Figure 7:**
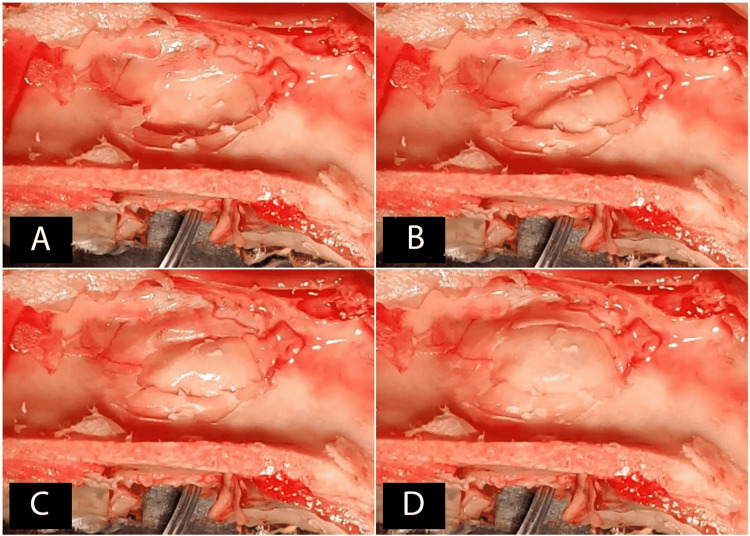
Membrane elevation using the controlled internal maxillary sinus elevation device (CIMSED), and monitoring the sinus membrane through the exposed medial wall of the maxillary sinus A: Applying the initial pressure. B: Membrane elevation observation. C: Increasing the applied pressure. D: Resulting membrane elevation

The maxillary sinus membranes were elevated using the CIMSED, starting at 10 mbar, and maintaining this pressure for five seconds, then increasing the pressure to 10 mbar for another five seconds until the sinus membrane started to be elevated (Figure 7A). The pressure increase with continuous monitoring (Figures 7B, 7C) was continued until reaching a height of 15 mm, perforating the membrane, or the pressure leakage was noticed (Figure 7D). The controlled hydrodynamic pressure was used to lift maxillary sinus membranes in six halves of sheep heads (Group 1) whereas the controlled pneumatic pressure was used to achieve the lifting in the other six halves (Group 2).

Outcome measures

The Height of the Maxillary Sinus

**Figure 8:**
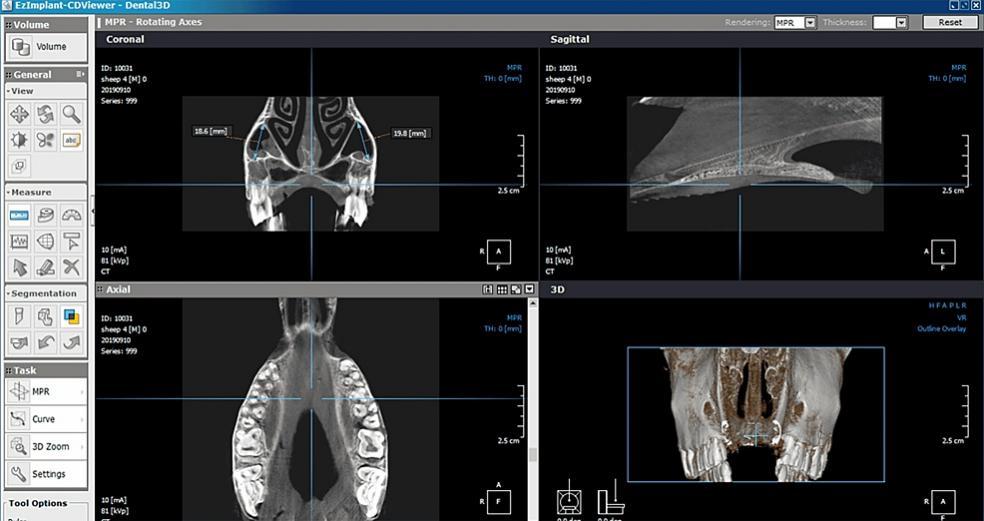
The measurement of the maxillary sinus height on the CBCT image CBCT: cone-beam computed tomography

Before the surgical procedures were started, the height of every maxillary sinus (observed on CBCT images) was measured at the third premolar, first molar, second molar, and third molar levels (Figure 8). The measurement was performed (on coronal view) from the lowest point of the sinus floor to the highest point of the sinus roof.

The Pressure Value

During the pressure application, the pressure values needed to start elevation of the maxillary sinus membranes were recorded in both groups.

The Elevation Height

**Figure 9:**
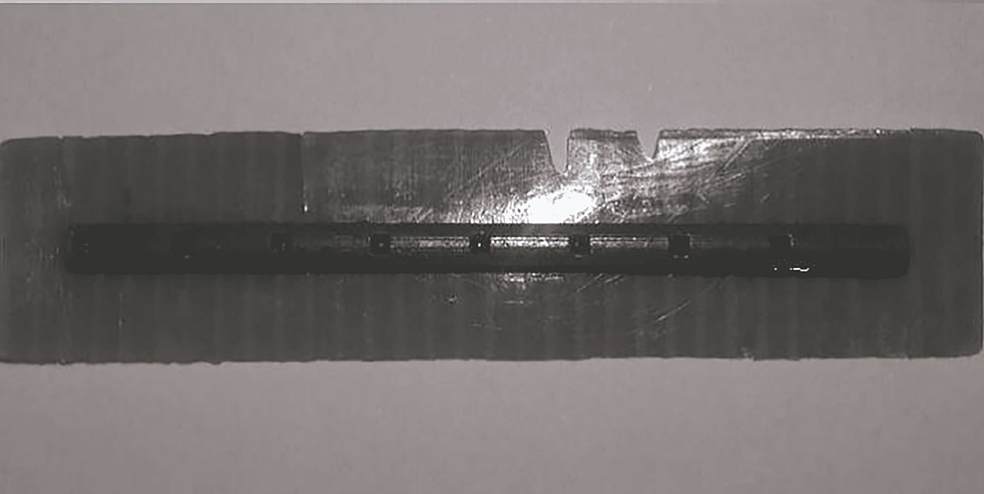
A special tool was used with a diameter of 2 mm, and a length of 40 mm This tool was designed and manufactured by the authors of this paper

After completing that application, the elevation height of every maxillary sinus membrane was evaluated and measured in both groups as well. This evaluation and measurement were achieved by inserting a special tool into the prepared bed. The special tool was designed and printed via a 3D printer with a diameter of 2 mm, and a length of 40 mm (Figure 9).

Detection of Perforation

The presence or absence of perforation was investigated visually.

Statistical analysis

The arithmetic means and standard deviations of the maxillary sinus heights, the pressure values, and the elevation amounts were calculated. Only descriptive statistics are provided in this report.

## Results

Twelve halves of sheep heads were included in the experimental procedures. The mean height of the maxillary sinuses in the collected sample at the third premolar, first molar, second molar, and third molar levels was 12.8 ± 5.6 mm, 22.2 ± 4.9 mm, 21.3 ± 5.2 mm, and 25.4 ± 3.6 mm, respectively (Table 1).

**Table 1:** Maxillary sinus height in the experimental units of the sheep heads

The experimental unit	Height of the maxillary sinus at the third premolar	Height of the maxillary sinus at the first molar	Height of the maxillary sinus at the second molar	Height of the maxillary sinus at the third molar
1	20 mm	22.9 mm	24.2 mm	25.5 mm
2	15 mm	24 mm	25.3 mm	24.1 mm
3	10 mm	16.4 mm	16.3 mm	22.8 mm
4	4.6 mm	15 mm	16 mm	22.8 mm
5	5.7 mm	21.8 mm	18.6 mm	28.2 mm
6	5 mm	19.9 mm	16.8 mm	26.77 mm
7	19.8 mm	30.3 mm	33.6 mm	31.4 mm
8	18.6 mm	25.4 mm	26.9 mm	28.2 mm
9	16.3 mm	29 mm	19.8 mm	20 mm
10	16.5 mm	26.1 mm	21 mm	19.5 mm
11	11.3 mm	17.7 mm	19.9 mm	26.6 mm
12	11.4 mm	18.3 mm	17.4 mm	29.1 mm
The mean ± standard deviation	12.8 ± 5.6 mm	22.2 ± 4.9 mm	21.3 ± 5.2 mm	25.4 ± 3.6 mm

The maxillary sinus membranes started to be elevated (by using the CIMSED) at a mean pressure of 21.6 ± 7.5 mbar when hydrodynamic pressure was applied (Table 2), and at a mean pressure of 23.3 ± 8.1 mbar when pneumatic pressure was applied (Table 3).

**Table 2:** The values of hydrodynamic pressure that were required to start elevation (Group 1)

The experimental unit in Group 1	The values of hydrodynamic pressure required to start elevation
1	20 millibar
2	30 millibar
3	10 millibar
4	20 millibar
5	30 millibar
6	20 millibar
The mean ± standard deviation	21.6 ± 7.5 millibar

**Table 3:** The values of pneumatic pressure that were required to start elevation (Group 2)

The experimental unit in Group 2	The values of pneumatic pressure required to start elevation
1	30 millibar
2	30 millibar
3	20 millibar
4	30 millibar
5	10 millibar
6	20 millibar
The mean ± standard deviation	23.3 ± 8.1 millibar

**Figure 10:**
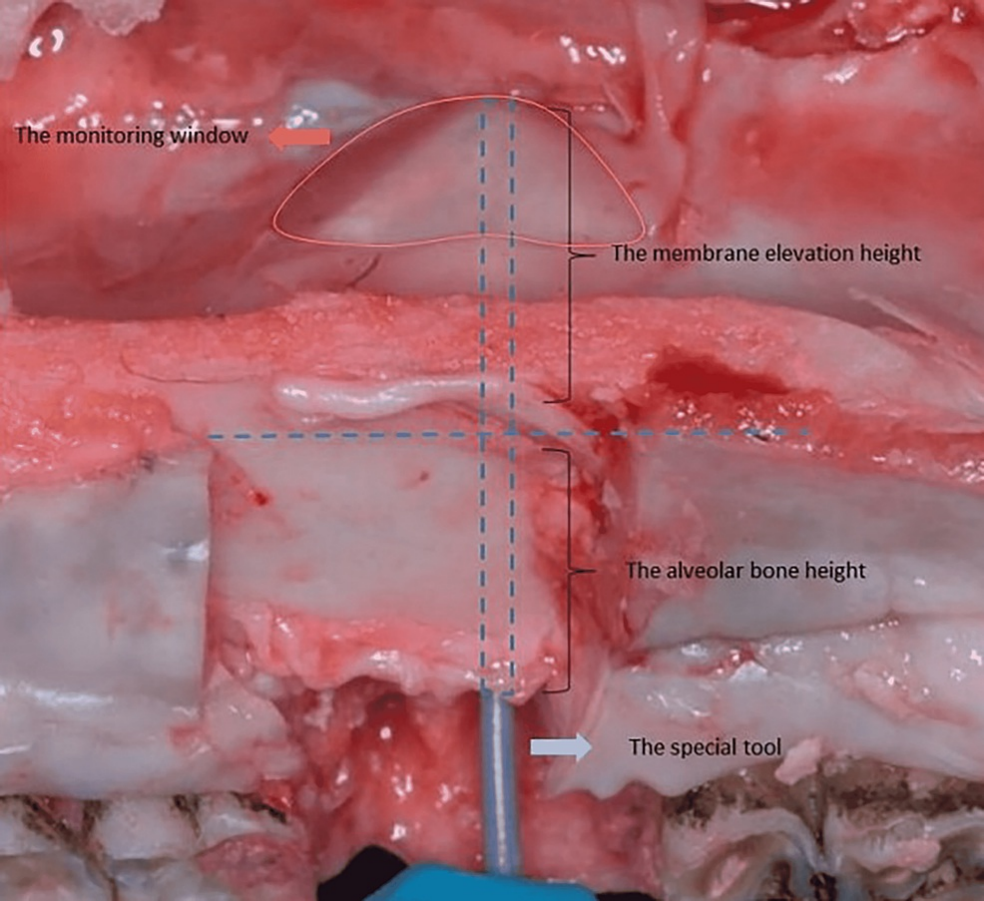
The evaluation and measurement of elevation height (mm) of the maxillary sinus membrane by using a special tool

Measuring the elevation height of the membrane was done manually by utilizing a special tool, and the results were obtained by subtracting the height of the alveolar bone from the fully measured length (Figure 10). The mean value of elevation heights of the sinus membrane after using controlled hydrodynamic (Group 1) and pneumatic pressure (Group 2) was 13.00 ± 2.76 and 10.33 ± 3.88 mm, respectively.

**Figure 11:**
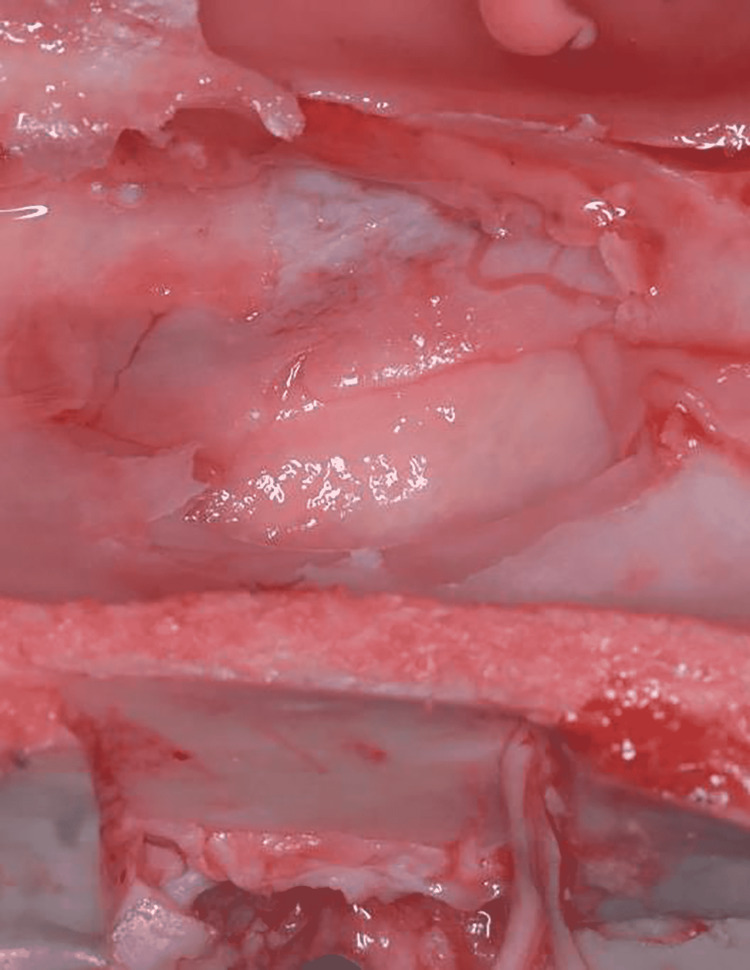
Visual inspection of perforations

The membrane was inspected visually through the monitoring window (Figure 11), and this was done during pressure application and after procedure accomplishment. No perforations were observed in either of the groups.

## Discussion

Maxillary sinus pneumatization, which occurs after posterior teeth extraction, results in the diminishing height and width of the alveolar bone [3]. This phenomenon renders the implantation process less predictable with more complications; therefore, several methods have been introduced to elevate the sinus membrane, and researchers have been striving to invest in more secure and predictable techniques [10]. In this study, in order to test the effectiveness of the maxillary sinus lift using controlled hydrodynamic or pneumatic pressure, a special device (CIMSED) was manufactured, and a particular method of pressure application was performed as well.

This study demonstrated the effectiveness of using controlled hydrodynamic, or pneumatic pressure, in internal maxillary sinus elevation in sheep, as the results were comparable to the results of external maxillary sinus elevation [19]. The mean value of the resulting elevation heights in Kim et al.'s and Jesch et al.'s clinical studies was 13.9 and 9.2 mm, respectively [14,20]. These results were close to our ex-vivo study findings, and this may be due to the application of hydrodynamic pressure directly to the maxillary sinus membrane through the alveolar bone in all previous studies. There was a marked difference between the results of our study and the results of Yassin Alsabbagh et al.'s study (which used the CAS kit system for lifting maxillary sinus membranes in sheep), where the maximum elevation height of the membrane was 5 mm [21]. The difference can be explained by the fact that the CAS kit system depends on the application of uncontrolled hydraulic pressure [13,21], unlike the technique used in our preliminary study.

The mean pneumatic pressure value needed to lift the maxillary sinus membrane was greater than the mean hydrodynamic pressure, as in Troedhan et al.'s study [16]. This may be due to the possibility of air particles compressing, and thereby dispersing a part of the applied pressure [16]. The previous phenomenon does not occur when using the pressure of non-compressible liquids such as saline used in this study [16,22]. This indicates that the use of controlled hydrodynamic pressure in the context of maxillary sinus elevation procedures is more predictable.

The results of this study confirmed the absence of perforations in the membranes in both techniques, which is in line with the findings of Sotirakis et al. [12] and Kim et al. [14]. The absence of perforations in this research may be a result of the suitable applied pressure, which was raised gradually and at small intervals. Moreover, the hydrodynamic and pneumatic applied pressures were not concentrated at certain points but distributed evenly on the maxillary sinus membrane [16,23].

The use of controlled hydrodynamic or pneumatic pressure may spare us the extensive surgical work required in the process of the external maxillary sinus elevation [19], as well as reduce the incidence of perforation of the maxillary sinus membrane, or even totally avoid it. However, the aforementioned techniques have significant implications for the success of grafting the maxillary sinus.

Limitations of the current work

The main drawback of testing such techniques in freshly slaughtered sheep was the leakage of pressure from the alveolar bone, and the inferior wall of the maxillary sinus. To minimize the leakage, the pressure was applied directly to the membrane of the maxillary sinus by the silicone tube that was sealed to the place of the implant by flowable composite, and the monitoring window on the medial wall of the maxillary sinus was minimized as much as possible. The CIMSED needs further mechanical adjustments if it is to be used in clinical studies in humans because of the inability to monitor sinus membrane changes as in this experimental study.

## Conclusions

This ex-vivo preliminary study showed that using controlled hydrodynamic or pneumatic pressure to elevate the maxillary sinus membrane internally could be effective. There was no membrane perforation after the elevation process. However, using sheep as an animal model for testing controlled techniques for maxillary sinus membrane elevation has some limitations. Testing more controlled procedures and developing new devices are required to advance the current methodology in maxillary sinus elevation operations.
